# The X-Ray Treatment of Skin Cancers, Etc.

**Published:** 1902-03

**Authors:** 


### The X-Ray Treatment of Skin Cancers, Etc.

In a recent number of the *Philadelphia Medical Journal* Dr. J. F. Rinehart praises highly the X-ray treatment of epithelial skin cancers and non-parasitic sycosis. In the X-ray treatment there is no pain, no scar and there is the possibility of a more thorough eradication of the disease than would be possible with the knife or caustic. He believes that the light and not the static electricity produces the effect of destroying the cancer cells. With Dr. W. A. Pusey he thinks that four classes of cases may be successfully dealt with by means of the X-rays: (1) Superfluous hairs may be removed; (2) diseased hairs in sycosis, tinea tonsurnss and favus may be removed; (3) inflammatory products may disappear by absorption from use of the X-ray; (4) tissues of low vitality, as in lupus, may be destroyed. R.

